# An Australian aged care home for people subject to homelessness: health, wellbeing and cost–benefit

**DOI:** 10.1186/s12877-023-03920-3

**Published:** 2023-04-28

**Authors:** Claire M. C. O’Connor, Roslyn G. Poulos, Anurag Sharma, Costanza Preti, Najwa L. Reynolds, Allison C. Rowlands, Kyall Flakelar, Angela Raguz, Peter Valpiani, Steven G. Faux, Michael Boyer, Jacqueline C. T. Close, Leena Gupta, Christopher J. Poulos

**Affiliations:** 1 Centre for Positive Ageing, HammondCare, Sydney, Australia; 2 School of Population Health, University of New South Wales, Sydney, Australia; 3 School of Psychology, University of New South Wales, Sydney, Australia; 4 Neuroscience Research Australia, Sydney, Australia; 5 Haymarket Foundation, Sydney, Australia; 6 Departments of Rehabilitation Medicine and Pain Medicine, St Vincent’s Hospital, Sydney, Australia; 7 Chris O’Brien Lifehouse, Sydney, Australia; 8 Prince of Wales Clinical School, University of New South Wales, Sydney, Australia; 9 Sydney Local Health District, Sydney, Australia

**Keywords:** Homelessness, Aged care, Trauma informed care, Wellbeing, Health, Service evaluation, Cost–benefit

## Abstract

**Background:**

Older people subject to homelessness face many challenges including poor health status, geriatric syndromes, and depression, coupled with barriers in accessing health and aged care services. Many are in need of formal aged care at a younger age than the general population, yet, in Australia, specialised aged-care services to support this vulnerable cohort are limited.

**Methods:**

This study was an evaluation of a new purpose-built aged care home for people with high care needs and who are homeless or at risk of homelessness. Over the first 12 months post-admission, the study examined: (1) changes in residents’ physical, mental, psychological and social health, and (2) the costs incurred by the study cohort, including any cost benefit derived.

**Results:**

Thirty-five residents enrolled in the study between March 2020 – April 2021. At admission, almost half of residents were within the range for dementia, the majority were frail, at high risk for falls, and had scores indicative of depression. Over time, linear mixed-effect models showed significant improvement in personal wellbeing scores, with clinically significant improvements in overall health related quality of life. Levels of physical functional independence, frailty, and global cognition were stable, but cognitive functional ability declined over time. Comparison of 12 month pre- and post- admission cost utility data for a smaller cohort (*n* = 13) for whom complete data were available, suggested an average per resident saving of approximately AU$32,000, while the QALY indicators remained stable post-admission.

**Conclusion:**

While this was a small study with no control group, these preliminary positive outcomes add to the growing body of evidence that supports the need for dedicated services to support older people subject to homelessness.

**Supplementary Information:**

The online version contains supplementary material available at 10.1186/s12877-023-03920-3.

## Background

Older people experiencing, or at risk of, homelessness in Australia and internationally face many challenges [1–3]. Compared to younger people who are homeless, older homeless people are more likely to experience functional limitations, have chronic health conditions and fewer social interactions, and these occur earlier than would be expected in non-homeless cohorts of the same age [4, 5]. In Australia, it is estimated that over 116,000 people are homeless, of whom 16% are older Australians (aged 55 years and over) [6, 7]. While males are disproportionately represented (63%) amongst older Australians experiencing homelessness, the proportion of older women experiencing homelessness is increasing (growing by 31% from 2011–16) [6]. Of the broader older Australian population (≥ 65 years), Indigenous Australians account for 0.9%, yet they represent 8% of the older homeless population [6]. For all older Australians who are homelessness or at-risk of homelessness, a better understanding around health and wellbeing, causes of homelessness, and the costs of addressing homelessness is vital [8]. In Australia, while older people who are homelessness or at-risk of homelessness are considered in policy [9], there are policy deficits when it comes to those with *high care needs*, as well as in the data sources required to inform policy for this group; for example, in the recent New South Wales (NSW) Department of Communities and Justice report on pathways to homelessness, only 4% of participants were > 55 years of age [10], which is far less than the estimated 16% of homeless older Australians [6, 7].

The international literature often describes poor health status, geriatric syndromes (e.g. falls, frailty, dysfunction in everyday living skills, cognitive impairment) and depression in the older homeless population. These conditions are reported for both long-term and newly homeless older individuals, and occur prematurely compared to older people in the non-homeless population [8, 11]. Frailty specifically has been associated with increased vulnerability, adverse outcomes, and mortality, and is an important outcome to measure when planning care [12]. In parallel, older homeless people have frequently experienced trauma and abuse during their lives and often face barriers in accessing health and aged care services, risking rapid decline in health with subsequent premature ageing and mortality [13–15]. As a consequence, many are in need of aged care at a younger age than the general population [2]. With such complex needs, specialised aged-care models are indicated, yet such services in Australia are limited [2]. While smaller, home-like care homes deliver improved quality of life for older people requiring residential care [16], for older people who are homelessness or at-risk of homelessness it is also important to incorporate a trauma-informed framework [2, 17, 18]. A trauma-informed approach considers a range of domains that may impact an individual (e.g. trauma exposure, social disadvantage, long-term homelessness, mental and co-morbid health conditions), and uses this context when providing care and support [2, 18, 19].

In Melbourne Australia, the ‘Wicking Project’ evaluated a specialised model of residential aged care over a series of two consecutive pilot studies (*n* = 14 in 2011 and *n* = 15 in 2016) to support residents living with a history of homelessness, cognitive impairment due to alcohol related brain injury, and high behaviour support, but low physical healthcare needs [20]. The specialised Wicking model of care included intensive case management, one-on-one care support, structured individualised activity programs and access to multidisciplinary support services as required. Positive outcomes were reported for reductions in depression, anxiety, and average alcohol consumption, along with increases in productivity as measured by the Community Integration Questionnaire [20]. Outcomes from this series demonstrated the feasibility of successfully transitioning a group of older people experiencing alcohol related brain injury from homelessness into specialised care, providing support for future studies to broaden outcomes to older homeless people in general.

Building on previous work described above, a new purpose-built aged care home for people with high care needs and who are homeless or at risk of homelessness was recently opened in Sydney, Australia in 2020. This service accommodates a mixed cohort of residents who either have experience of homelessness or are considered to be ‘at-risk’ (defined in footnotes of Table 2) of homelessness [21], therefore, the term ‘subject to homelessness’ will here on in be used to describe the cohort in this study (meaning they are currently affected by or it is possible they will be affected by homelessness). Inner Sydney was selected as the site for the new care home as it has a uniquely high proportion of older individuals subject to homelessness [22] and needed local accommodation options [23].

With a total capacity of 42 residents, the home, which is split across four floors, with one specifically for women and another for residents with higher-care needs (physical and/or cognitive), aims to provide a non-institutional, trauma-informed approach to care through both design and operation. The building features: private bedrooms with ensuite bathroom; rooms designed with a ‘transition space’ before entering common areas to respect possible trauma and mistrust faced by people with a history of homelessness; fully functioning domestic kitchens and laundries on each floor, allowing autonomy for residents to use all parts of the home at a time of their choosing. A multi-skilled care worker-led staffing model, supported by registered nurses and other health care professionals, fosters development of a relationship of trust between these front-line care staff and residents, and also minimises the need for ‘strangers’ (such as cleaners, kitchen staff, maintenance and delivery personnel) to enter the home.

The purpose of this study was to evaluate the outcomes from this new service to help inform policy and practice for older people with high care needs subject to homelessness. The study aimed to examine: (1) changes in residents’ physical, mental, psychological and social health from admission up to 12 months post admission; (2) the costs, primarily to government, incurred by the study cohort in the 12 months leading up to, and in the 12 months following, their admission to the care home, including any cost benefit derived.

## Methods

### Study design

This study is an evaluation of a new purpose-built residential aged care home in Sydney, Australia, for people who are subject to homelessness. A longitudinal design was used to explore: resident health and wellbeing; and cost–benefit, primarily to government, derived from the care home over the first 12 months.

Health and wellbeing measures were collected at baseline (within the first month; M = 22 days, 95%CI 13.9 – 30.7 days), 6 months (M = 6.4 months, 95%CI 6.2 – 6.7), and 12 months (M = 12.3 months, 95%CI 11.9 – 12.7) post admission. Data on the cost of utilisation of health and human services over the 12 months prior and up to 12 months post admission to the home were collected via self-report of residents, contact with previous service providers, and linking hospital records. Specifically, at baseline, researchers guided participants through a purpose-made survey (Additional file 1) to prompt residents to remember which services they had contact with during the 12 months prior to admission (e.g. ambulance, health and hospitals, justice, housing, generalist and specialist community services). The research team then followed up with each identified service to determine the extent of each contact (e.g. hospital admissions, length of stay, number of contacts with homelessness services, nights in Government housing etc.). Hospital records were accessed from three Local Health Districts in Sydney, NSW. Participants gave informed consent prior to any hospital or service records being accessed.

### Participants and setting

Participants were recruited for the evaluation between March 2020 – April 2021. All new residents admitted to the care home as a permanent resident during the study period were invited to participate in the service evaluation; residents admitted for respite only were excluded. Inclusion criteria required that residents demonstrated a willingness and ability to provide informed consent and a willingness and capacity to participate in and comply with study data collection.

The study was approved by the St Vincent’s Hospital Sydney Human Research Ethics Committee (2019/ETH11898), with separate Site-Specific Approvals (*n* = 3) received for each hospital or Local Health District where resident hospital data extraction was required. Written informed consent was obtained from all participants.

### Instruments

Outcome measures (described briefly below, with measure and administration details summarised in Table 1) were collected by researchers who were not involved in providing care or other services within the home.

**Table 1:** Outcome measures with associated administration notes and collection time points

Domain	Measure	Measure details	Collection time point(s)	Administration notes
Cost-utilisation	Self-report survey, contact with service providers, hospital records	See Additional file 1 for details	Baseline 12-months	Researcher interview with resident; researcher follow-up (email and/or phone) with services and hospitals
Functional independence	Australian Functional Measure (AFM): motor functional independence; cognitive functional independence	17 items that are summed to provide two domains: motor functional independence scored out of 84 (12 items covering self-care, sphincter control, transfers, locomotion), and cognitive functional independence scored out of 35 (5 items covering communication, social cognition). Each item is scored on a scale from 1 (total assistance required) to 7 (complete independence), accounting for what the resident is capable of doing in terms of physical ability, mental health, cognition and behaviour [24, 25]	Baseline 6-months 12-months	Researchers interviewed care staff directly involved with care of each specific resident
Cognition	Rowland Universal Dementia Assessment (RUDAS)	Scored out of 30, the scale includes six items covering: memory, visuospatial orientation, praxis, visuo-constructional drawing, judgement and language. Scores of 22 or less are indicative of potential dementia or cognitive impairment [26, 27]. High test–retest (ICC 0.98) and interrater (ICC 0.99) reliability [26], and good internal consistency (Cronbach’s α = 0.80; [28])		Researchers administered the RUDAS directly with each resident
Frailty	Clinical Frailty Scale (CFS)	The scale ranges from 1—‘very fit’ to 9—‘terminally ill’ where a higher score indicates a higher degree of frailty. A score of 6 indicates ‘moderate frailty’ [29, 30]. Very good interrater reliability (weighted kappa 0.86, 95%CI 0.84–0.87 [31];)		Researchers interviewed care staff directly involved with care of each specific resident
Mobility	Timed Up and Go test (TUG)	The TUG involves measuring the time (secs) taken to stand up from a seated position, walk three metres, turn around, and return to the seated starting position. A score ≥ 16.5 s indicates reduced mobility and a greater likelihood of falling [32]. High test–retest (ICC 0.91) and interrater (ICC 0.91) reliability in residential care [33]		Administered by the research team with each resident who was physically able
Mental health	Geriatric Depression Scale (GDS)	15-items. Higher scores are indicative of greater likelihood of depression, with a cut-off score ≥ 5 suggesting the presence of depression [34, 35]. Moderate internal consistency (Cronbach’s α = 0.75; [36])		Residents were provided the form to self-complete their ratings, however the majority requested the researchers to administer the scales in interview form
	PTSD Checklist – Civilian	17-items. Respondents rate how much they have been bothered by each listed PTSD symptom from 1 – ‘not at all’ to 5 – ‘extremely’. Scores range from 17–85, with higher scores indicating greater likelihood of PTSD. With an estimated pooled prevalence rate of PTSD in homeless populations of 27% [37], we selected 36–44 as the cut-off score for individuals in this study [38, 39]. High internal consistency (Cronbach’s α = 0.94) and good test–retest reliability (ICC 0.92) [40]		
Subjective wellbeing	Personal Wellbeing Index-Adult (PWI-A)	Satisfaction across 7 life domains is measured on a scale from 1 – ‘no satisfaction at all’ to 10 – ‘completely satisfied’: standard of living, health, achieving in life, relationships, safety, community connectedness, future security. Each domain is converted to a standard score from 1–100 and then averaged across domains to determine an overall subjective wellbeing score, also known as ‘personal wellbeing index’ [41]. Higher scores indicate better subjective wellbeing; scores between 51–69 indicate personal wellbeing that is likely to be ‘challenged’ or ‘compromised’, and scores ≥ 70 suggest a ‘normal’ level of wellbeing [42]. The Australian national index of subjective wellbeing is 75.1 [43]. Moderate to good internal consistency (Cronbach’s α = 0.70–0.85; [41])		
Overall health related quality of life	EuroQol-5 Dimension (EQ-5D) Visual Analogue Scale (VAS)	Participants indicate their perceived health on a scale from 0 – ‘worst health you can imagine’ to 100 – ‘best health you can imagine’. Mean VAS scores according to a South Australian norms project were 78.6 (65–74 years) and 72.7 (75 + years) [44]. Sufficient test–retest reliability [45]		Researchers provided the scale, and residents indicated their score
Cost–benefit	EQ-5D-5L system	Comprises 5 dimensions: mobility, self-care, usual activities, pain/discomfort and anxiety/depression [46]. Each dimension has 5 levels: no problems (level 1), slight problems, moderate problems, severe problems and extreme problems (level 5). The person is asked to indicate their health state by ticking the box next to the most appropriate statement in each of the five dimensions. There are 3,125 possible health states defined by combining one level from each dimension, ranging from 11,111 (full health) to 55,555 (worst health). Excellent test–retest reliability at the index score level [47]		Residents were provided the form to self-complete their ratings, however the majority requested the researchers to administer the scales in interview form

*Functional independence* was measured using the Australian Functional Measure (AFM) as used in the Australian National Aged Care ‘Resource Utilisation and Classification study’ (RUCS) [24]. The AFM is based on the Functional Independence Measure [48] but differs in that the ‘stairs’ item has been removed, and it reports what a person *can* do, rather than what they are observed doing. *Cognition* was measured using the Rowland Universal Dementia Assessment (RUDAS) [26]. *Frailty* was assessed using the Clinical Frailty Scale (CFS) which involves the use of clinical judgement to measure fitness and frailty in older people [29]. *Mobility*: was assessed using the Timed Up and Go test (TUG) [49]. *Mental health* was assessed with the Geriatric Depression Scale (GDS) to screen for depression [34]. To account for the high prevalence of trauma experience in homeless populations, the self-report PTSD Checklist – Civilian was used to determine whether participants meet DSM-IV symptom criteria for post-traumatic stress disorder [38]. *Subjective wellbeing* was measured using the Personal Wellbeing Index-Adult (PWI-A) [41]. *Overall health related quality of life* was measured using the EuroQol-5 Dimension (EQ-5D) Visual Analogue Scale (VAS), which is a standalone portion of the EQ-5D and has been recommended for use in conjunction with the EQ-5D-5L rating system [50, 51]. *Cost–benefit* was evaluated using the EQ-5D-5L system [52]. EQ-5D-5L health states are converted into a single index ‘utility’ score using a scoring algorithm based on public preferences. In this study, the UK value set and scoring algorithm were used to calculate utility scores as an Australian scoring algorithm is not yet available for the 5L [53].

### Data analyses

*Impact of frailty on health and wellbeing*: Frailty has been associated with adverse outcomes such as increased disability, greater healthcare dependency, hospitalisation and death [54, 55]. As frailty is a risk factor for adverse outcomes, in addition to evaluating change in frailty over the course of the study, we undertook a comparison of two groups of residents according to their baseline Clinical Frailty Scale score (‘not frail’ – scores of 0–5, and ‘frail’ – scores of 6–9); this breakdown of frailty scores has been used previously to predict adverse outcomes [56].

Statistical analyses were conducted using SPSS 26.0 (Windows). Baseline demographics were analysed descriptively for the entire resident cohort (Table 2). To analyse differences between frailty groups, Shapiro–Wilk tests indicated skewed baseline data for the majority of the measures (AFM, RUDAS, CFS, PTSD, PWI, EQ5D-VAS), therefore non-parametric measures were used for pairwise comparisons (Mann–Whitney *U*); *X*^2^ tests were used for dichotomous comparisons [57]. The number of resident participants who died during the study period was compared between frailty groups (*X*^2^ tests) and hospital use (emergency department [ED] presentations, hospital admissions, length of stay) within the first 12 months of living in the home was examined between frailty groups (Mann–Whitney U tests). To explore changes in health and wellbeing measures in residents over time, linear mixed-effect models were used. This approach accounted for the missing data [58] that occurred over each of the timepoints from baseline, 6 months and 12 months as residents either died, dropped out, or declined to answer specific questionnaires. While the specific number of responses differed according to outcome measure, the number of residents participating in each round of data collection declined over the three timepoints as follows: *n* = 35, *n* = 27, *n* = 18. Due to the small sample size, longitudinal analyses were conducted with the cohort as a whole. Time was the only fixed effect in the model. A random intercept was included in each model as individual resident baseline variability was the only included random effect. The variability of any estimated parameters was determined by both the random and fixed effects in the model. For each of the following dependent variables, a separate model was built: AFM Motor, AFM Cognitive, RUDAS, CFS, TUG, GDS, PTSD-C, PWI, and EQ5D-VAS. The analysis applied a linear first-order polynomial due to the small sample size.

**Table 2:** Resident baseline demographics

	**All residents** **(*n* = 35)**
Median age at admission—yrs (range)	75.6 (67–81)
Sex (F/M)	12/23
Homelessness history^a^ n (%)	
Experience of homelessness	13 (37.1%)
At-risk	22 (62.9%)
Housing prior to admission n (%)	
Government housing	20 (57.1%)
Residential aged care	7 (20%)
Crisis accommodation, rough sleeping, boarding house	5 (14.3%)
Private rental, independent living	3 (8.6%)
Referral source n (%)	
Hospital/Social worker	22 (62.9%)
Residential Aged Care	6 (17.1%)
Home Care provider	3 (8.6%)
Homelessness service	3 (8.6%)
Other (friend)	1 (2.9%)
Referral reason^b^ n (%)	
High care – health	19 (54.3%)
High care – cog, psych, drug	14 (40%)
Social	2 (5.7%)

^a^For homelessness history, residents were assigned to one of two groups: (1) experience of homelessness: including rough sleeping (e.g. living in improvised dwellings, tents, or sleeping out), staying in supported accommodation for the homeless, couch surfing or temporarily staying with other households, living in a boarding house or temporary lodging, or living in a dwelling that is severely crowded [6]; (2) at-risk for homelessness: residents actually had a history of housing, but experience poverty and may experience precarious or insecure tenure. These people are often socially isolated with limited or no contact with family, and often also have health issues [21]. For the purposes of this evaluation, included in the ‘at-risk of homelessness’ group were residents who had been living in Department of Housing accommodation, private rentals, or were transferred from another nursing home

^b^Reason for referral was scored according to one of three categories: social (e.g. domestic violence; no family support; not happy at previous nursing home), high care needs due to health status (e.g. declining health; unable to manage health independently; functional decline), and high care needs due to cognitive, psychological or drug-related support needs (e.g. unable to manage independently due to cognitive decline such as dementia, psychological needs; self-neglect). For residents who had more than one referral reason, the category identified as the primary reason by the clinical team at the time of referral to the home was selected

*Cost-utility analysis*: A cost-utility approach was used from the perspective of the government as funder, as almost all of the costs incurred in supporting the cohort pre- and post- admission to the care home can be attributed to Federal or State governments. Changes in quality of life (EQ-5D-5L; [46] were compared to 12 month pre- and 12 month post- admission costs related to the utilisation of healthcare services, justice system, public housing and temporary accommodation, community care and utilisation of other homelessness agencies. As this study was an evaluation of an operating service, there was no opportunity or research funding capacity to employ a control group. A detailed costing study was done by which utilisation of healthcare services in the 12 months pre- and post- admission was converted to dollars where individual utilisation data were available (ED visits, hospital inpatient admissions, utilisation of other healthcare services). For example, for hospital inpatient episodes, diagnosis related groups and length of stay for each episode was combined with the national efficient price reported by the Independent Hospital Pricing Authority [59] to estimate the funding provided by the government for that episode. Similarly, Medicare benefit schedule fee data [60] were used to cost general practitioner, medical specialist, outpatient and other diagnostic services such as CT scans. The utilisation of non-health services (such as public housing, justice system, police visits, home care packages [61], other homelessness services) was costed using publicly available data or data from individual service provider cost estimates.

The overall cost of care for the aged care home for 12 months for all residents was provided by the operator of the aged care home, and included the value of all government subsidies, including homelessness supplements. Resident contributions (set at 85% of the aged care pension; [62]) for living in the aged care home were not included in the cost of care within the home on the assumption that residents’ would have incurred a similar living cost if they were not residing in the care home. As per the attrition reported for the health and wellbeing data, complete 12-month pre and 12-month post cost-utility data were not able to be collected for all residents; therefore, *n* = 13 residents who had a complete cost-utility data set were included in this portion of the analysis.

## Results

**Fig. 1:**
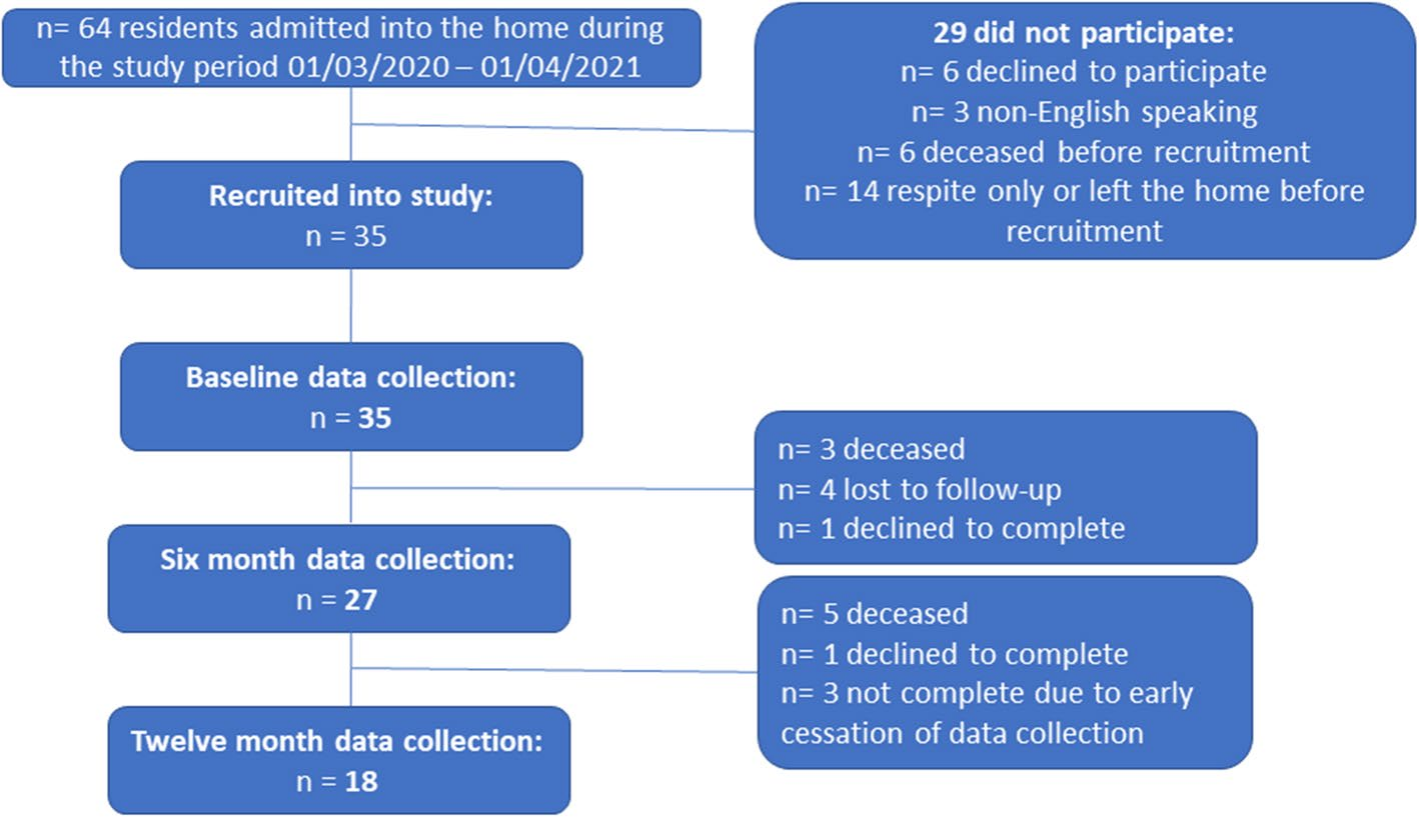
Recruitment and retention of participants over the 12-month study period

A rolling recruitment period occurred as residents came into the home, with data collected from March 2020 to November 2021. During the recruitment period, 64 residents were admitted to the home; of these, 29 did not participate in the evaluation (Fig. 1). The majority (48.3%) of those who did not participate had been admitted to the home as respite only, so were excluded from the study; the remainder either declined to participate in the evaluation (20.7%), died before being recruited (20.7%), or were excluded due to being non-English speaking (10.3%). Due to delays with admissions to the home associated with the COVID-19 pandemic, recruitment of participants into the study was significantly delayed. Therefore, to facilitate timely data analysis, the last three participants that were recruited did not complete 12-month assessments. Ultimately, 35 residents agreed to participate and were recruited into the study; they had a median age of 75.6 years and the majority were male (65.7%). Most participants were referred via hospital (usually by a social worker), with the primary referral reason being high health care needs. Just over a third of participants had experienced homelessness in their life, while the remainder were considered at-risk of homelessness. For most participants (77%), the most recent accommodation prior to admission was either government housing or another residential aged care home (Table 2).

### Baseline

**Fig. 2:**
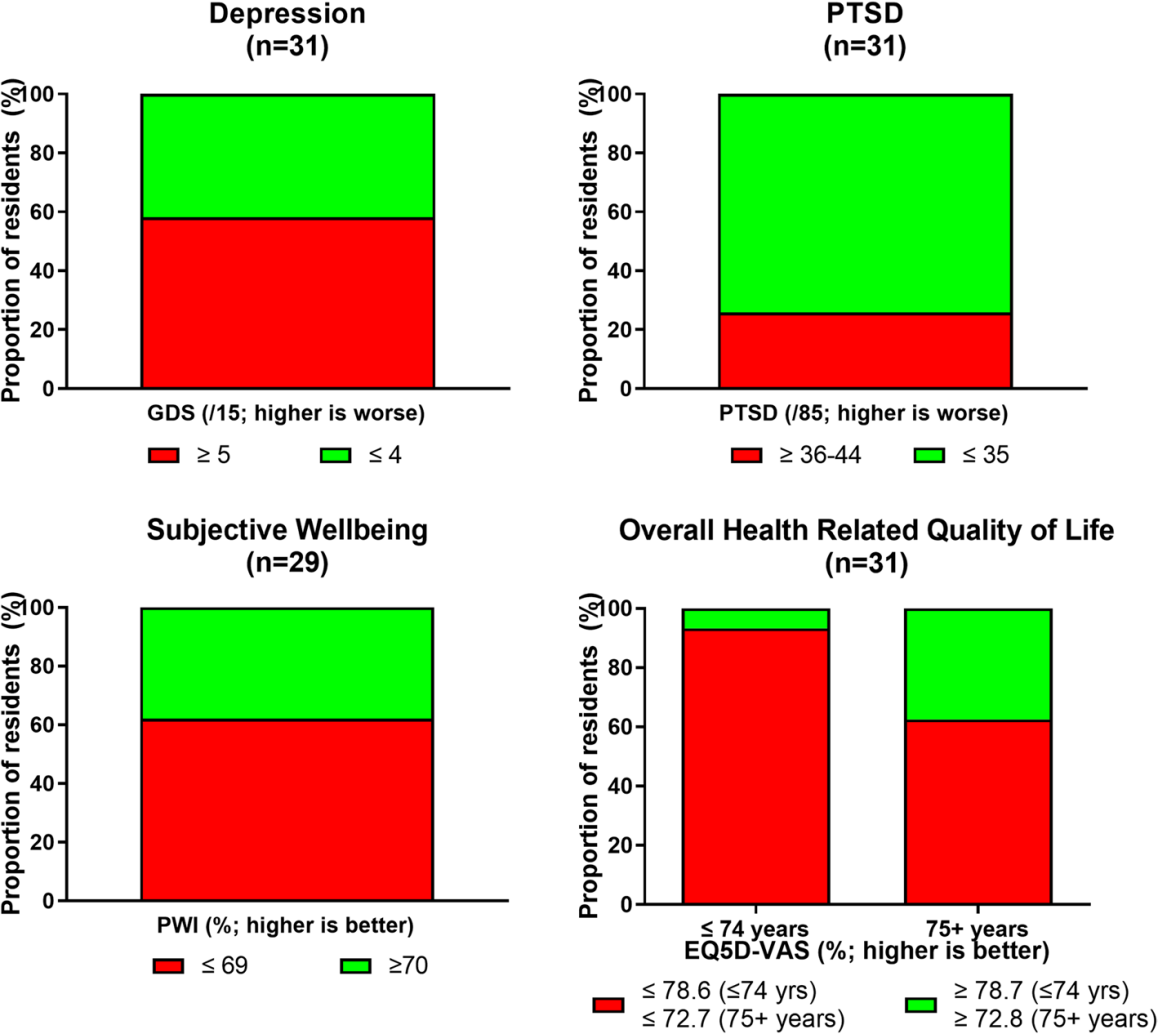
Proportion of residents according to specified cut-off scores for physical and cognitive performance measures at baseline. AFM—Australian Functional Measure (median RAC resident scores from RUCS study: motor function = 43 and cognitive function = 19 [24]); CFS – Clinical Frailty Scale (RAC scores from RUCS study: 7-Severly frail = 31%; 6-moderately frail = 23%; 5-mildly frail = 15%; [24]); RUDAS – Rowland Universal Dementia Assessment (≤ 22 cut-off for dementia or cognitive impairment [26]); TUG – Timed Up and Go (≥ 16.5 s indicates reduced mobility and greater likelihood of falling [32])

The majority of residents were moderately frail (CFS; [29], at high risk for falls (TUG; [49], and had scores suggesting the presence of depression (GDS; [34]). While median scores for PTSD did not reach the cut-off for homeless populations, a quarter of residents did fall above this cut-off (Fig. 2) [38, 39]. Residents rated their subjective wellbeing (PWI) and health-related quality of life (EQ5D-VAS) well below Australian and aged-based norms, with the vast majority of younger residents in particular scoring below the respective cut-off [43, 44] (Table 3).

**Table 3:** Resident baseline health and wellbeing data

Outcome measure	Median score for all residents (*n* = 35)
AFM Motor (/84; higher is better) ≤ Median score RAC = 43 [24]	59 (36–77)
AFM Cognitive (/35; higher is better) ≤ Median score RAC = 19 [24]	31 (25–35)
RUDAS (/30; higher is better) ≤ 22 cut off for cognitive impairment [26]	23.5 (19–27)
CFS (/9; higher is worse) 5 – mildly frail 6 – moderately frail 7 – severely frail	6 (5–7)
TUG (Secs; higher is worse) ≥ 16.5 s indicates reduced mobility [32]	30 (20–50)
GDS (/15; higher is worse) ≥ 5 cut off for depression	6 (3–8)
PTSD (/85; higher is worse) ≥ 36–44 cut off for PTSD	27 (22–36)
PWI (%; higher is better) ≥ 70 suggest a ‘normal’ level of wellbeing [42]	60 (52–74)
EQ5D-VAS (%; higher is better) ≤ Mean score 65–74 yrs = 78.6 ≤ Mean score 75 + yrs = 72.7 [44]	50 (50–70)

Scores are medians with 25^th^ -75^th^ percentiles

*AFM Motor* Australian Function Measure Motor Scale, *AFM* *Cognitive* Australian Function Measure Cognitive Scale, *CFS* Clinical Frailty Scale, *EQ5D-VAS* Euroquol5D Visual Analogue Scale, *GDS* Geriatric Depression Scale, *PTSD* Post Traumatic Stress Disorder Checklist Civilian, *PWI* Personal Wellbeing Index Adult version, *RAC* Residential Aged Care, *RUDAS* Rowland Universal Dementia Assessment, *TUG* Timed Up and Go

**Fig. 3:**
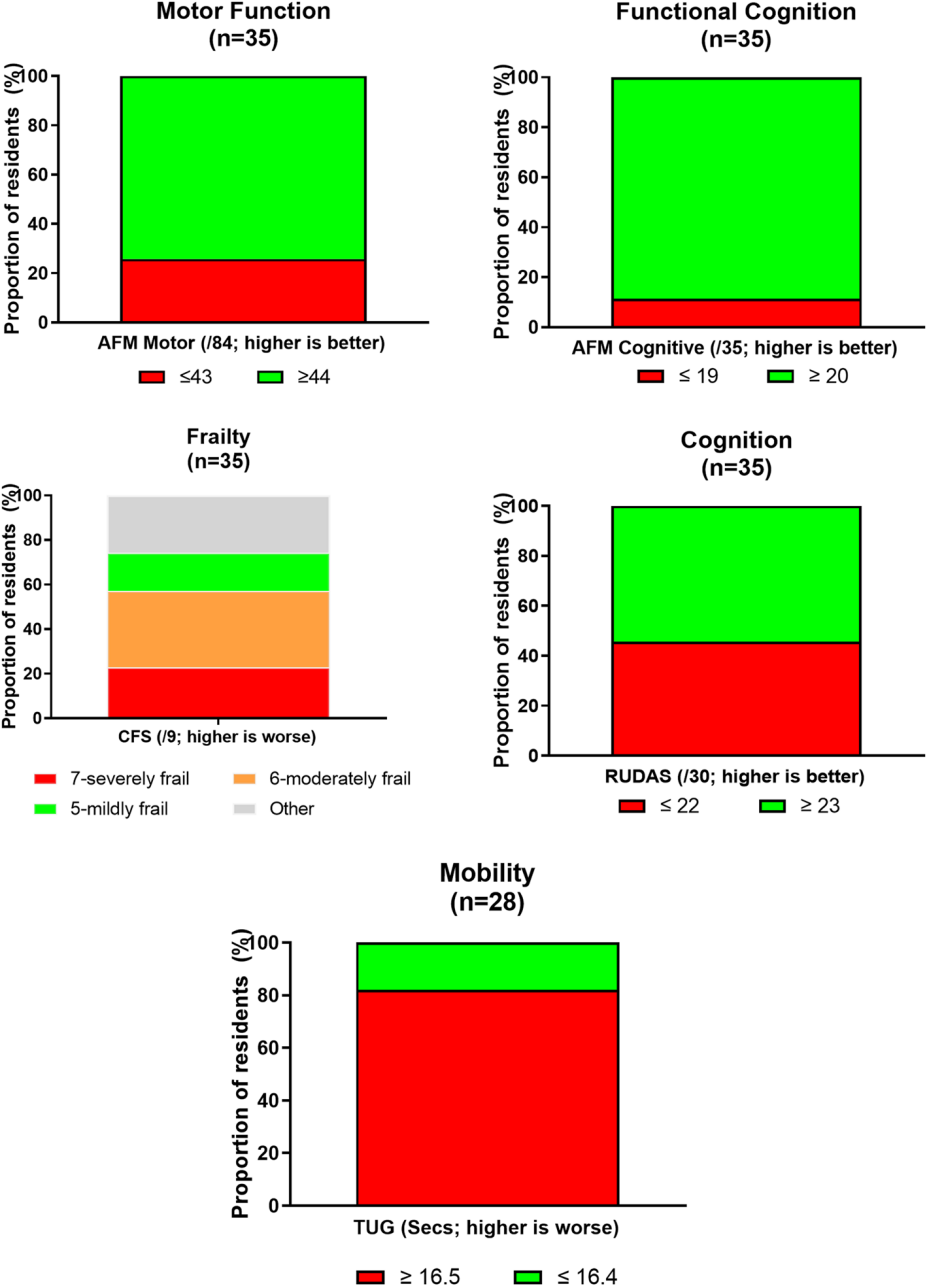
Proportion of residents according to specified cut-off scores for mental health and wellbeing measures at baseline. EQ-5D-VAS—EuroQol-5 Dimension Visual Analogue Scale (Mean VAS scores according to a South Australian norms project were 78.6 (65–74 years) and 72.7 (75 + years) [44]); GDS—Geriatric Depression Scale (≥ 5 suggests depression [34, 35]); PTSD–Civilian—Post-Traumatic Stress Disorder Civilian Checklist (cut-off score set at 36–44 [38, 39]); PWI—Personal Wellbeing Index (≥ 70 suggest a ‘normal’ level of wellbeing [42])

Pairwise comparisons (based on CFS scores) confirmed that residents in the ‘not frail’ group performed better than the ‘frail’ group on physical functioning measures: AFM Motor (median ‘not frail’ = 80, ‘frail’ = 52; U = 27.5, *p* < 0.001), TUG (median ‘not frail’ = 21, ‘frail’ = 40; U = 47.0, *p* < 0.05). No other differences between groups were identified and the two groups were similar in age, sex distribution and baseline cognitive score. At admission, median scores for all residents were within the range for mild cognitive impairment, with almost 46% of residents falling below cut-off, suggesting dementia or cognitive impairment (Fig. 3) (RUDAS; [26, 27].

### Frailty group differences in deaths and hospital use over the study period

For hospital use in the first 12 months after admission to the home, there were no differences between frailty groups, after Bonferroni correction, for ED presentations, hospital visits, hospital length of stay, or outpatient occasions of service. In contrast, the rate of death differed between groups during the first 12 months post admission; no residents in the ‘not frail’ group died compared to 42.9% of residents in the ‘frail’ group (*X*^2^ = 8.077, *p* < 0.005) (Table 4).

**Table 4:** Hospital service use and deaths over the first 12 months post admission per frailty group

	Not frail (*n* = 14)	Frail (*n* = 21)	Test statistic	*p*-value
ED # of presentations	54	44	132.5^a^	NS
Hospital # of admissions	19	39	114.5^a^	NS
Hospital length of stay (days)	62	230	89.0^a^	= .042^c^
Outpatient # occasions of service	117	51	136.5^a^	NS
# residents deceased	0	9	8.077 ^b^	< .005

^a^Mann–Whitney U tests

^b^*X*^2^ test

^c^No longer significant after applying Bonferroni adjustment for multiple comparisons

### Longitudinal changes for the full cohort

**Fig. 4:**
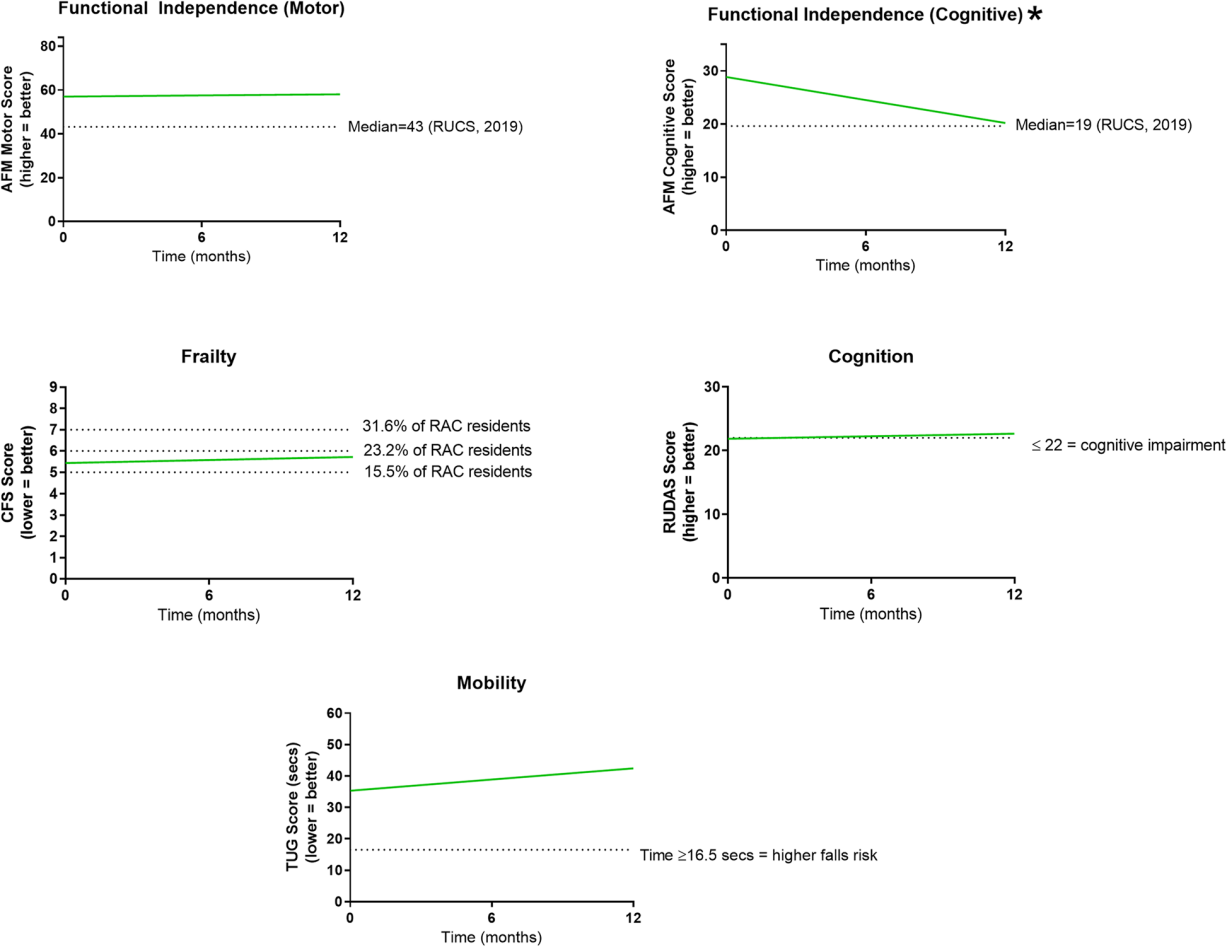
Model representations of performance on physical and cognitive performance measures over time. AFM—Australian Functional Measure (median RAC resident scores from RUCS study [24]); RAC – Residential Aged Care; RUDAS – Rowland Universal Dementia Assessment; TUG – Timed Up and Go. Plots are modelled score representations; * *p* = 0.01. The dashed line on each graph represents the respective reference or cut-off score from each measure; Frailty (% of RAC resident scores from RUCS study; [24])

Linear mixed-effect models showed that over time, there was no change in motor functional independence, level of frailty, mobility, or global cognition (RUDAS). In contrast, cognitive functional independence (AFM-Cognitive) declined (*F*_1,53.07_ = 7.08, *p* = 0.01) (Fig. 4).

**Fig. 5:**
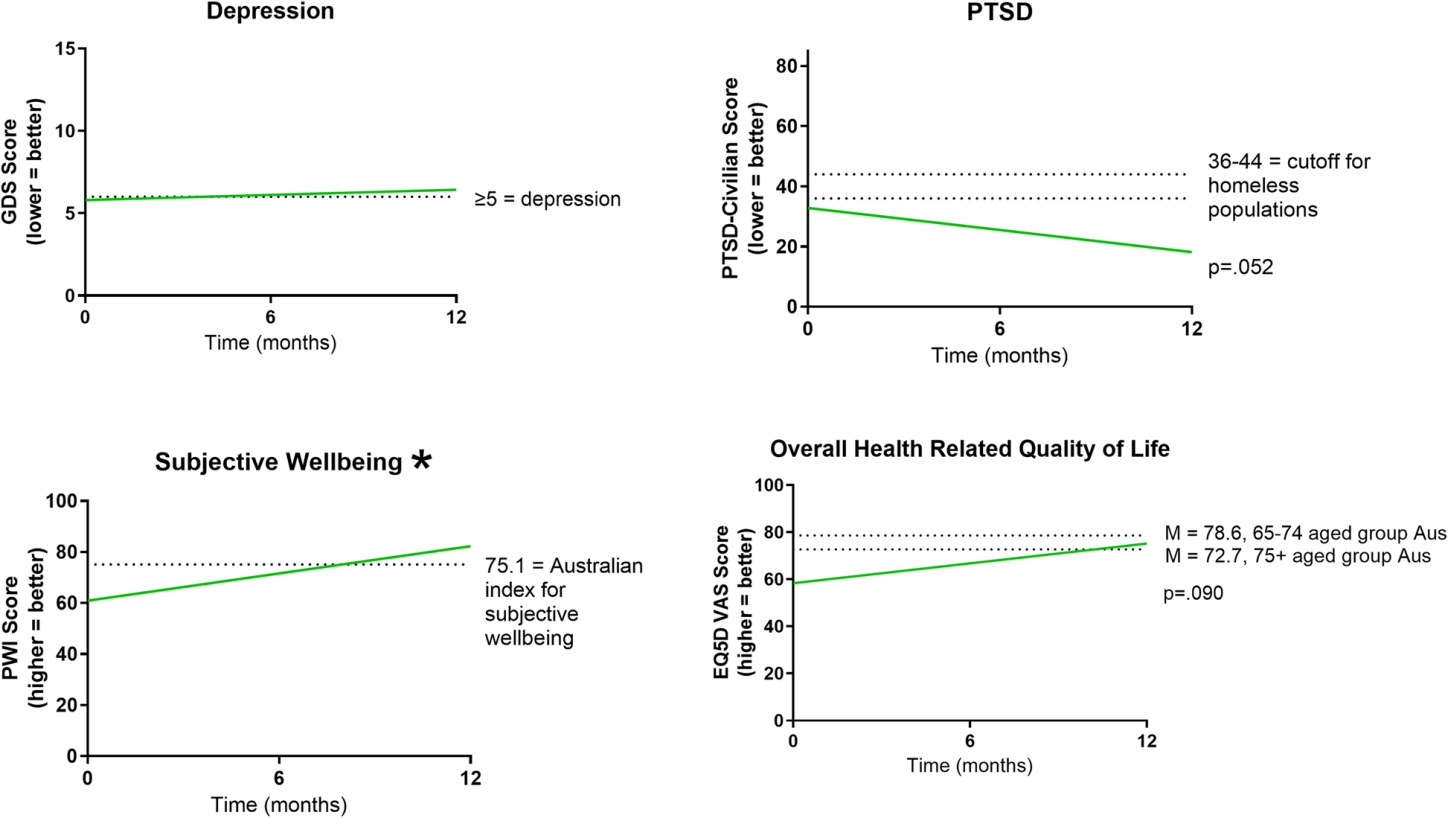
Model representations of performance on mental health and wellbeing measures over time. EQ-5D-VAS—EuroQol-5 Dimension Visual Analogue Scale; GDS—Geriatric Depression Scale; PTSD–Civilian—Post-Traumatic Stress Disorder Civilian Checklist; PWI—Personal Wellbeing Index. Plots are modelled score representations; * *p* < 0.05. The dashed line on each graph represents the respective reference or cut-off score from each measure

Modelling showed significant improvement in personal wellbeing scores over time (PWI; *F*_1,36.18_ = 5.16, *p* < 0.05), so that by 12 months, scores had moved from a stage of being ‘challenged/compromised’ to being above the Australian index for subjective wellbeing (Fig. 5). There was also a trend for improvement in overall health related quality of life (EQ5D-VAS; *F*_1,42.70_ = 3.01, *p* = 0.09) mirroring the wellbeing improvements identified with the PWI. In addition, resident EQ5D-VAS scores improved on average by 16.8 points over the first 12 months living in the home. Finally, while no differences over time were identified for depression, there was a trend for improvement in PTSD (*F*_1,33.58_ = 4.06, *p* = 0.052); importantly, this change is clinically significant as modelled scores improved on average by 14.6 points over the year (Fig. 5).

### Cost–benefit outcomes

In comparison to the improvements observed in the EQ5D-VAS scores (reported above), there was no significant overall change (p-value 0.6) in EQ-5D-5L scores over the 12 months. At baseline, the median EQ-5D-5L score was 0.542 with the minimum being just 0.178. Out of 13 individuals, EQ-5D-5L scores increased slightly for seven individuals and decreased slightly for six individuals.

A comparison of cost of healthcare utilisation 12 months prior to and 12 months post admission to the care home shows a reduction in costs, with the most prominent decrease through reduction in inpatient hospital episodes. For example, in the cohort of 13 individuals with complete cost-utility data who participated in the 12-month follow up survey, the average per capita cost to the government from inpatient episodes decreased from an average of AU$22,300 prior to admission to AU$10,400 post entry to the aged care home. Similarly, per capita cost to the government from ED visits decreased from an average of AU$4,600 to AU $1,600. Overall it was observed that total per capita cost to the government decreased by an average of AU $32,000 for this cohort once the older person subject to homelessness was admitted to the aged care home over the 12-month period, with a combined per resident average cost of AU $153,068 in the 12 months prior to admission, compared to AU $121,101 for the 12 months post-admission. Virtually all of this cost reduction is attributable to government funders, both Federal and State governments.

## Discussion

This study reported the health, wellbeing and cost–benefit outcomes for a group of residents over the first 12 months of living in a new aged care home specifically for people subject to homelessness. The study found that residents overall, reported improvements in aspects of mental wellbeing, with other outcomes demonstrating stable levels of physical functional independence, frailty, and global cognition, but with a deterioration in cognitive functional ability, over the 12 months following admission into the home. A per capita saving in the cost to government, not associated with any decline in resident-reported quality of life, is also reported, however this finding is limited to the subset of 13 residents for whom complete cost-utility data were available. It is noted that the study was conducted during the COVID-19 pandemic, and that the care home, as with most others in Australia, experienced periods of ‘lock-down’ and restrictions on resident activities [63].

In line with previous research into older people subject to homelessness [15], upon admission to the home, more than half of resident participants had mild depression, just less than half had cognitive impairment, and more than half had moderate or severe physical frailty. Overall, this evaluation suggests that, for this cohort of older people who were subject to homelessness, moving into a purpose-designed aged care home with a specialised model of care [2] generated positive mental wellbeing outcomes. Residents reported a significant improvement in their subjective wellbeing so that by 12 months modelled scores were higher than the Australian index for subjective wellbeing. This was mirrored by clinically significant improvements in overall health-related quality of life (EQ5D-VAS), which by 12 months was within Australian age-based norms [44], and in PTSD, which by 12 months was well below cutoff for homeless populations [37, 39]. Additionally, improvements in EQ5D-VAS scores over the first 12 months living in the home is more than double a previously reported minimal clinically important difference estimate of 8 points [64]. These outcomes suggest that the supportive environment of the new aged care home, with its trauma-informed approach to care [2], has had a positive impact on resident wellbeing, despite the compounding influence of a global pandemic. This inference aligns with previous research that has discussed the benefits of trauma-informed care, ‘wraparound services’ and regular assistance from support workers for older people subject to homelessness [18, 65–67]. Similar to the finding relating to EQ5D-VAS scores, improvement in PTSD scores over the first 12 months of living in the home is more than double the reliable change index [68] reported for this scale [69]. It is possible that the permanent, supportive environment of the home with social support from staff implementing trauma-informed care also contributed to residents reporting fewer PTSD symptoms [18, 70].

In contrast to the other improvements in wellbeing, resident scores indicating the presence of depression did not change over the first 12 months. This reflects previous Australian research that described high rates of cognitive impairment and mental health conditions in a cohort of older homeless individuals in Sydney [22]. Overall, our residents exhibited cognitive impairment, frailty, and depression, which have been associated with greater likelihood of having additional geriatric syndromes in older homeless individuals [71], and this extends to greater risk of premature morbidity and mortality [54]. When we separated the residents according to level of frailty, this was demonstrated in the frail group who experienced significantly higher levels of mortality than the not frail group, mirroring recent research [55] that also illustrated differences in mortality between frailty groups. Additionally, just over a fifth of residents died prior to being recruited for the study, more than double a previously reported mortality rate of 7.9% within the first 30 days post admission [72].

More than 60% of residents in our study were referred to the home from hospital. Older people subject to homelessness who are discharged from hospital require specific aged care support options [73]. It is clear that older individuals subject to homelessness require dedicated services that specialise in both homelessness and aged care domains [2, 23]. A randomised controlled trial involving middle-aged and older prefrail homeless women in the USA found that a health promotion program that addressed frailty in addition to psychological and social issues such as alcohol and drug use was beneficial [74]. In Australia, the Wicking project demonstrated positive outcomes from a specialised model of residential aged care to support residents with a history of homelessness and alcohol related brain injury, showing significant reductions in depression, anxiety, and average alcohol consumption [20]. The positive outcomes in resident wellbeing from the present study provides further support for the need for specific services to support older people subject to homelessness. Tailored services should provide a holistic approach incorporating positive physical environments (i.e. private resident rooms each with ensuite bathrooms and central domestic kitchens and living areas on each floor) and a model of care that applies a trauma-informed approach [18, 19] that recognises the likely trauma associated with experience of homelessness as well as social disadvantage, substance dependence and mental health, and uses this context to address any health conditions [2].

The Australian National Aged Care ‘Resource Utilisation and Classification study’ (RUCS) provided a snapshot of the health and wellbeing of residents from 30 sites within the Australian residential aged care sector across the east coast of Australia [24]. In comparison to the RUCS data, our participants were younger, but they were equally as frail upon admission to the home, which aligns with research that people who are subject to homelessness are at risk for physical frailty, regardless of age [71, 75]. In contrast to the RUCS data that described an increase in frailty over time within residential aged care [24], there was no overall change in frailty in our sample. Further, at the time of admission to the home, the residents in our study demonstrated higher levels of functional independence in both motor and cognitive domains when compared to RUCS study participants, and consistent with the frailty findings, physical functional independence was also able to be maintained over 12 months.

These findings of the maintenance of physical functional ability are also at variance with studies reporting on the impacts of institutionalisation and COVID-19 on older people. Throughout the COVID-19 pandemic, older adults have experienced declines in physical activity and in turn reductions in strength and balance [76–78]. Specifically, within residential aged care settings in Australia, lockdowns, visitor restrictions and staff shortages associated with the pandemic have resulted in functional, physical, cognitive and psychological declines in residents [63]. Also, as the main focus of residential aged care generally is to support residents within the context of their functional limitations, rather than trying to actively restore function [79], residential care environments may have an ‘institutionalising’ influence whereby the staff are “doing” things for residents that they would have previously managed independently [80, 81]. It is possible that for the cohort in this study, of whom around 65% had transitioned from more independent living arrangements (i.e. Government housing units, boarding houses, or private rentals), a residential care home with a specialised model of care [2] and improved nutrition through access to regular home-style meals, counteracted some of the impacts of the pandemic or the institutionalisation that affect general aged care cohorts [82, 83].

Cognitive functional independence (as measured by AFM) in our participants declined to the point that, by 12 months, mean scores were similar to those in the RUCS participants, but the maintenance of global cognition (as measured by RUDAS) suggests that other factors may be at play. Cognitive functioning in our participants may have been impacted by impaired social cognition, behaviour profiles and mental health conditions that frequently exist in a cohort of older people subject to homelessness [22, 84, 85]. Brief cognitive screening measures, such as the RUDAS, may not capture the range of cognitive dysfunction common in homeless populations, such as impairments in social cognition [86, 87]. Indeed, the majority of residents had been living independently prior to admission to the home, so moving into a new environment and cohabiting with other residents may have been challenging for some. Also, as the AFM is a proxy measure, it is possible that at baseline, the residents had not yet integrated and staff had not observed many inter-resident interactions prior to scoring the AFM-Cognitive. By 6 and 12 months however, staff had observed more interactions between residents, which also means scoring at this point may have been subject to observer bias [88]. Additionally, once people have moved into a nursing home, they may not have the opportunity to engage in complex everyday activities that should be considered when scoring the AFM. Therefore, if residents had not engaged in specific activities once at the home, it would be difficult for staff to objectively provide a score for that resident [89, 90].

A lower cost to government in the 12 months post admission into this residential aged care home, associated with no deterioration in EQ-5D-5L score, indicates improvement in efficiency (i.e., achieving the same outcome with lower costs) and is a cost-effective strategy. While there are no directly comparable reports of cost-effectiveness evaluations of similar care homes specifically for older people subject to homelessness, the findings from the Wicking Project did report an annual saving per resident of AU $11,000 [20]. People subject to homelessness incur greater costs to government than the general population, as shown in a recent NSW government report into people who accessed specialist homelessness services [10]. This report revealed that the average cost to government per person over six years was AU $186,000, rising to AU $706,000 for the 5% of the cohort with the highest costs (or, almost 4 times on average, or up to 14 times, respectively, the cost to government of age matched controls). However, older people (60 + years) were extremely under-represented in this report, comprising only 2% of the cohort.

There are several study limitations. The care home studied targeted older people subject to homelessness, which includes people who were either homeless, or who were ‘at-risk’ of homelessness [21]. This could make direct comparison with other research into the profiles and needs of the older homeless population less reliable. The small sample size and the fact that this study evaluated a single specialised aged-care home in Sydney, mean that outcomes might not be generalisable to other aged care facilities or other cohorts of older people subject to homelessness. The AFM and CFS were completed via proxy by the care staff with support from the research team, whereas in the RUCS study, the AFM was only completed by clinically experienced allied health professionals [24]. Also, inter-rater, and test-re-test were not assessed; thus there may have been reliability limitations impacting these proxy-rated measures [91]. Given the improvement trends identified for PTSD and health related quality of life, it is possible that the small sample size contributed to a type II error, and a larger sample may have reached statistical significance. In addition, the fact that 42.9% of residents identified at baseline as ‘frail’ died within the first 12 months of admission into the home, compared with no deaths in the ‘not frail’ group, may mean that the overall positive findings on mental wellbeing and lack of decline in physical functional ability may be impacted by a survivor bias. Other sources of bias could include participation bias regarding any differences between the residents who agreed to participate in the evaluation and those who did not participate. It is to be noted that our study uses a pre-post analysis. While the care provided may have led to decreased healthcare utilisation costs, it is also possible that the study cohort had incurred higher than usual healthcare utilisation costs (e.g. inpatient hospital episodes and ED visits) in the 12 months prior to care home admission, and thus cost savings post-admission might be due to regression towards the mean phenomenon [92].

Given the paucity of specialised aged care homes catering specifically to older homeless populations, it was not possible to use more rigorous approaches to evaluation, such as clustering. That being said, the current study builds on the outcomes from the Wicking Project [20], which also had a small sample size (*n* = 4); an added strength of the current study was use of the linked cost and utilisation data. Finally, the impact of the COVID-19 pandemic resulted in some delays in recruitment and data collection for the study, and may have potentially impacted on resident experiences throughout the study period. For example, lockdowns meant that there were periods of time were residents were unable to see family or participate in their usual community activities, which may have impacted on their perceived wellbeing or quality of life.

## Conclusions

The residents in this study reported improved mental wellbeing and maintained physical functioning over the first 12 months of living in this new aged care home. The study also suggests a reduction in the cost to government, however this finding should be viewed with caution and further research is needed. While this was a small study with no control group, these preliminary positive outcomes add to the growing body of evidence that supports the need for dedicated services to support older people subject to homelessness. Greater understanding around the experiences of residents, staff and the homelessness sector around the new home will contribute to development of future services that meet the needs of the sector. More work is needed to identify preventative intervention points and ultimately reduce the number of older people who experience or are at risk of homelessness. In the meantime, this study shows that there is great potential to support the older population subject to homelessness through tailored service provision, with wellbeing benefits for the people themselves and financial benefits to society more broadly.

## Supplementary Information

**Additional file** **1.**

## Data Availability

The data that support the findings of this study are available from the corresponding author upon reasonable request.

## Abbreviations

AFM: Australian Functional Measure
CFS: Clinical Frailty Scale
ED: Emergency Department
EQ-5D: EuroQol-5 Dimension
GDS: Geriatric Depression Scale
NSW: New South Wales
PTSD-C: Post-Traumatic Stress Disorder-Civilian
PWI-A: Personal Wellbeing Index-Adult
RUCS: Resource Utilisation and Classification study
RUDAS: Rowland Universal Dementia Assessment
TUG: Timed Up and Go test
VAS: Visual Analogue Scale
