# Supplementary material for: An Australian aged care home for people subject to homelessness: health, wellbeing and cost–benefit
Source: BMC Geriatr. 2023 Apr 28;23:253. doi: 10.1186/s12877-023-03920-3 (PMC10139912; doi:10.1186/s12877-023-03920-3)
Supplement: Supplementary file 1 — Additional file 1. [file 12877_2023_3920_MOESM1_ESM.docx]

**Service use survey**

This survey is to be used as a guide to assist residents to remember which services they may have used over the previous 12 months. Only relevant items are to be addressed – not all categories/items will be relevant for each resident. In speaking with the resident, service names will be identified, and the consent for release of information form will be personalised to ask for specific consent to release of information for each resident.

| **Service Type - broad** | **Service types - detailed**  (Tick all that apply and if resident is able to remember record service/agency name in the next column) | **Specific services identified by resident**  (List names of each specific service identified by the resident) | **Occasions of service/frequency;**  **Length of Stay for in-patient/residential services**  (For each identified service, record details on usage over past 12 months) |
| --- | --- | --- | --- |
| Health | ED (SVHA and/or other hospital)  In-patient including mental health admissions (SVHA and/or other hospital)  SVHA Homelessness Health  Aboriginal Medical Services (AMSs)/A&TSI Health Services  Dental Services  Drug and Alcohol Service (detox, residential, outpatient/community)  Mental Health Services  General Practitioner, including GP services within Homeless Services  Rehabilitation Services (incl. brain injury services)  Other specialist health services (HIV, Women’s Health, Sexual Health etc.) |  |  |
| Ambulance | Emergency transport to hospital  On-site/street-side care |  |  |
| Justice | Options include:  Number of police interactions (all types), or  specific interaction types e.g. offences, charges, infringement notices and move-on notices  Court appearances  Legal aid consultations  Imprisonment/detention (if relevant)  Victim of crime (Victims Support Package $)^[[1]](#endnote-1)^ |  |  |
| Housing | Specialist Homelessness Services (crisis, medium term, transitional and supported accommodation bed nights)  Social/community housing via FACS/ NGO (rent)  Case management (non-residential support) |  |  |
| Generalist community services | Emergency financial assistance  Meals, laundry, personal care services  Centrelink interviews/payment adjustments  Generic counselling/psychosocial support  Community Transport  Neighbourhood Centres/Advice Centres |  |  |
| Specialist community services | ATSI  CALD  LGBTIQ  Prison release support  Vietnam Veterans’ Counselling Service  Trustee and Guardian Specialist Support Team |  |  |

1. <https://www.victimsservices.justice.nsw.gov.au/Pages/vss/vs_financial_support/vs_financial_support.aspx> [↑](#endnote-ref-1)
